# Flexible and efficient triboelectric nanogenerators based on PVDF and boron nitride composite yarns and mats

**DOI:** 10.1039/d5nr05178a

**Published:** 2026-06-04

**Authors:** Sunija Sukumaran, Ahmadreza Moradi, Piotr K. Szewczyk, Urszula Stachewicz

**Affiliations:** a Faculty of Metals Engineering and Industrial Computer Science, AGH University of Krakow 30-059 Krakow Poland ustachew@agh.edu.pl

## Abstract

Flexible and scalable energy-harvesting materials are the driving force behind the emerging era of wearable and self-powered electronics that seamlessly integrate into modern life. Hence, triboelectric nanogenerators (TENGs) offer a versatile solution for integrating energy generation into smart textiles. Here, electrospun poly(vinylidene fluoride) (PVDF) mats and yarns incorporating boron nitride (BN) nanoparticles (1, 3, 5, and 10 wt%) were fabricated and characterized. The 5 wt% BN/PVDF composite exhibited the highest β-phase content and crystallinity, owing to the role of BN as an efficient nucleating agent that facilitates β-phase crystallization through strong interfacial interactions between the nanofiller and PVDF matrix. The triboelectric output was systematically compared across different structural configurations, including electrospun mats, yarns, and rolled-mat geometries. The BN/PVDF yarn-based TENG delivered the highest power density of 303 ± 0.30 mW m^−2^, representing ∼113% enhancement over pristine PVDF yarn and superior to previously reported PVDF-based devices. Moreover, despite its smaller active area, the yarn device produced higher power density than the mat counterpart (297 ± 0.43 mW m^−2^). These findings demonstrate that BN incorporation and yarn-based architecture enhanced power generation, providing a scalable route toward high-performance, flexible nanogenerators for wearable and self-powered electronics.

## Abstract 2

. BN-incorporated PVDF yarn TENGs deliver ~113% higher power density than pristine PVDF and outperform mats, offering a scalable route toward self-powered smart textiles and wearable energy harvesting.

## Graphical abstract

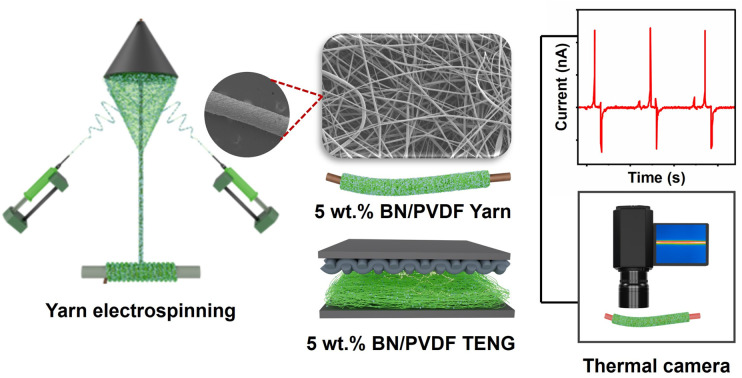

## Introduction

The growing integration of intelligent electronics into daily life requires sustainable and renewable energy sources that can reliably power portable and wearable systems.^1–3^ Among the various energy harvesting technologies, triboelectric nanogenerators (TENGs) have gained remarkable attention as highly promising mechanical energy harvesters, owing to their simple fabrication, material adaptability, and superior energy-conversion efficiency.^1,4^ TENGs, which utilize the coupling effect between triboelectrification and electrostatic induction to transform mechanical energy into electrical output, enabling self-powered operation of microelectronics and wearable sensors.^5–12^ Despite significant progress, the power output of TENGs remains insufficient for many practical applications.^13,14^ To address this challenge, various approaches have been proposed to enhance the surface charge density of triboelectric layers through material selection,^15^ surface modifications,^16,17^ and device structure optimizations.^18,19^ Nonetheless, many of the reported strategies involve complex fabrication processes, sophisticated device architectures, and expensive equipment, resulting in high production costs and limited feasibility for large-scale applications.^20,21^ In this regard, the use of triboelectric materials with high electron affinities and simple, scalable processing techniques offers a viable pathway toward efficient and cost-effective TENGs.^1,22^

High-performance TENGs rely on selecting suitable triboelectric pairs, commonly using nylon,^23^ cellulose,^24^ or polyvinyl alcohol (PVA)^25^ as tribopositive materials and poly (vinylidene fluoride) (PVDF),^6^ polydimethylsiloxane (PDMS),^26^ and polytetrafluoroethylene (PTFE)^27,28^ as tribonegative materials. Among these, PVDF stands out for its high electronegativity and piezoelectric properties.^29–31^ Several methods, including mechanical stretching, electrical poling, electrospinning, and the incorporation of fillers, have been employed to enhance the electroactive β-phase in PVDF, the phase primarily responsible for its superior piezoelectric response.^32–34^ Combining the scalable electrospinning process with low-cost, low-toxicity nanoparticles, such as zinc oxide (ZnO), barium titanate (BaTiO_3_), or graphene oxide (GO), represents a sustainable route to efficient TENGs.^2,6,31^ However, boron nitride (BN) nanoparticles offer a unique advantage over traditional oxides or conductive additives, providing high electrical insulation, thermal stability, superior piezoelectricity, mechanical robustness, and chemical inertness.^35,36^ Their inclusion within electrospun PVDF matrices thus provides an effective route to high-performance, flexible, and wearable TENGs.^2^

Beyond improving electrical properties, BN nanoparticles also impart high thermal conductivity (180–200 W m^−1^ K^−1^) to PVDF, facilitating efficient heat dissipation while maintaining electrical insulation.^37,38^ Such multifunctionality is particularly suitable for wearable TENGS, where managing localized heat is crucial during continuous operation.^39^ Consequently, BN/PVDF composites represent an attractive material for smart textiles that combine reliable triboelectric output with effective thermal regulation. However, most studies have focused mainly on film and electrospun mat structures, which lack the flexibility and textile compatibility essential for wearable devices.^40,41^ Yarn-based architectures overcome these limitations, providing high mechanical strength and can be woven or knitted onto fabrics.^23,42^ Nevertheless, the role of BN in tuning the triboelectric behaviour of PVDF yarns is yet to be studied, representing a critical opportunity to advance scalable, textile-integrated energy-harvesting technologies.

Herein, we report the fabrication of electrospun BN/PVDF composite mats and yarns to elucidate the role of BN nanoparticles on the thermal and triboelectric characteristics. Core–shell yarn-based TENGs were constructed using copper wire as the conductive core and electrospun PVDF and BN/PVDF fibers as the shell for triboelectric characterization. Similarly, thermal characteristics were evaluated using yarns prepared on resistance wire cores coated with PVDF and BN/PVDF fibers. By varying the BN nanoparticle concentration, the effects on fiber morphology, β-phase content, crystallinity, and triboelectric output were examined. Thermal responses were characterized through thermal camera imaging. Incorporation of BN enhanced β-phase and crystallinity, leading to improved triboelectric output, with the BN/PVDF yarn demonstrating slightly higher power density relative to the mat configuration. Also, mat-based structures are difficult to integrate into textiles, whereas yarn structure can be seamlessly integrated into fabrics. These results highlight the potential of BN/PVDF yarn as a far more suitable candidate for wearable and self-powered electronic textiles.

## Materials and methods

### Solution preparation and electrospinning

PVDF pellets (*M*_w_ = 275 000 g mol^−1^, Alfa chemistry Holbrook, NY, USA) were dissolved in a 1 : 1 (wt/wt) mixture of dimethylacetamide (DMAc, analytical standard, Avantor, Poland) and acetone (analytical standard, Avantor, Poland) solvents with continuous magnetic stirring at 650 rpm on a hot plate (IKA RCT basic, Germany) at a constant temperature set to 55 °C. A homogeneous 24 wt% PVDF solution was obtained after 4 h of constant stirring, following the previously reported protocol.^43,44^ BN (particle size <150 nm, density: 2.29 g cm^−3^, Sigma-Aldrich, UK) /PVDF composite solutions with different loading percentages of BN (1, 3, 5, and 10 wt%), were prepared by first dispersing the BN particle in DMAc and acetone 1 : 1 (wt/wt) ratio *via* ultrasonication (Emag, Emmi-E20, Germany) for 2 h. Then, the BN suspension is blended with the PVDF solution and stirred at 55 °C for 3 h to ensure uniform mixing. A final ultrasonication step was done for 2 h to further enhance the homogeneity of the composite solution prior to electrospinning.

Electrospinning of PVDF and BN/PVDF composite solutions was performed using an electrospinning equipment (SKE, Italy) with a climate control chamber. The electrospinning of PVDF was initiated by applying a positive voltage of +20 kV to a stainless-steel needle (outer diameter: 0.8 mm; inner diameter: 0.5 mm), with the distance between the needle tip and the grounded collector maintained at 18 cm with a polymer solution flow rate of 1 mL h^−1^. Electrospinning was conducted under controlled environmental conditions, with the temperature and relative humidity (RH) maintained at 25 °C and 30%, respectively, for both PVDF and its composite samples. The electrospinning parameters used for BN/PVDF composites with varying BN content (1, 3, 5, and 10 wt%) are summarized in Table S1 of the SI. The samples were labelled as PVDF M, 1, 3, 5, and 10 wt% BN/PVDF M, respectively.

The electrospun mats were deposited onto aluminum (Al) foil for scanning electron microscopy (SEM) and TENG characterization.

Yarn electrospinning: PVDF and 5 wt% BN/PVDF were selected to prepare the core–shell structured yarns (5 wt% BN shows the highest β phase and crystallinity, see result and discussion section). The samples were named PVDF Y and 5 wt% BN/PVDF Y. For thermal camera imaging, the yarn consisting of a resistance wire (200 µm diameter, RD 100/0,2 Block, Germany) coated with nanofibers as a shell was produced using a customized electrospinning system equipped with a yarn module (TechNOVA, China). For TENG, the core–shell yarns were prepared using a copper wire (diameter of 100 µm) as the core and PVDF or 5 wt% BN/PVDF as the shell. The process schematic of the yarn fabrication setup and the corresponding photograph of the produced yarn were presented in Fig. S1a and b (SI). Also, we have provided a visual demonstration of the yarn fabrication process presented in Video S1 in the SI. During yarn preparation, positive and negative voltages of 17 and 15 kV, respectively, were applied to the needles containing the polymer solutions. The distances from the positive and negative needles to the vortex collector were maintained at 12 cm and 10 cm, respectively. Polymer solution flow rates were set to 0.06 mL min^−1^, and yarn electrospinning was conducted at a temperature of 24 °C and 36% RH. The vortex and the collecting mandrel were operated at 200 rpm and 8 rpm, respectively, to facilitate uniform fiber deposition around the resistance wire.

Similarly, the core-shell yarns with a copper wire core were produced under the same electrospinning conditions described above, except for the needle-to-collector distances and mandrel rotation speed. The distances were increased to 16 cm (positive) and 18 cm (negative), and the mandrel rotation speed was set to 12 rpm. All remaining parameters were kept constant to ensure comparable core shell yarn formation.

### SEM, FTIR, and DSC characterizations

#### Scanning electron microscopy (SEM)

The morphological characteristics of the electrospun fibers were examined using SEM (Merlin Gemini II, Zeiss, Germany) operated at an accelerating voltage of 3 kV and a working distance of 5.8 mm. Before imaging, all samples were sputter-coated with an ∼8 nm thick layer of gold using a rotary pump sputter coater (Q150RS, Quorum Technologies, UK). Fiber diameters (*D*_f_) were measured from the SEM micrographs using ImageJ software (version 1.53c, USA). The distribution of BN nanoparticles within the composite fibers was examined by energy-dispersive X-ray spectroscopy (EDS, Bruker, Germany). Prior to analysis, the samples were sputter-coated with an ∼8 nm-thick gold layer using a rotary pump coater (Q150RS, Quorum Technologies, UK). Elemental mapping was performed for 300 s at an accelerating voltage of 10 kV, a current of 1 nA, and a working distance of 8.1 mm, using a backscattered electron detector.

#### Fourier transform infrared spectroscopy (FTIR)

FTIR spectroscopy was employed to identify the crystalline phases of PVDF and quantify the relative β-phase content. Measurements were performed using an Attenuated Total Reflectance Fourier Transform Infrared Spectroscopy (ATR-FTIR, Nicolet iS5, Thermo Fisher Scientific, USA) with a germanium crystal. Spectra were recorded over the wavenumber range of 600–1800 cm^−1^ with a resolution of 1 cm^−1^, averaging 64 scans per sample to ensure spectral accuracy and reproducibility.

#### Differential scanning calorimeter (DSC)

Thermal characterization of the samples was conducted using a differential scanning calorimeter (DSC, Mettler Toledo, Columbus, OH, USA). Measurements were performed in a dynamic Ar atmosphere over a temperature range of 25 to 250 °C at a heating rate of 10 K min^−1^, with the samples placed in Al crucibles.

### Thermal camera measurement

Thermal camera measurements were conducted following a previously established protocol.^45,46^ A yarn length of 15 cm was selected, and Joule heating was induced by applying an electrical current through the resistive wire until the standard reference tape reached 80 °C. The surface temperature distribution of the yarns was recorded using an infrared thermal camera (FLIR T560, USA), with a reference tape (emissivity = 0.96) attached to the yarn to ensure consistent temperature across all samples. Thermal data were analyzed using FLIR Tools software, extracting average surface temperatures from line profiles from three individual yarn samples. All thermal imaging experiments were carried out under controlled ambient conditions (23–25 °C and 24–25% RH).

### Triboelectric measurements

Triboelectric energy harvesting performance of PVDF and 5 wt% BN/PVDF mats was assessed *via* a bespoke setup equipped with a linear motor (LinMot P04, USA) operated in contact separation mode. The system operated by repeatedly tapping triboelectric-positive nylon fabric (commercially available) against triboelectric-negative PVDF M and 5 wt% BN/PVDF M. PVDF and BN/PVDF mats (12 mm in diameter) were mounted onto an Al electrode using silver paste (Acheson Silver DAG 1415M, China). The nylon fabric was similarly affixed to the Al electrodes using carbon tape. Tapping parameters were set to a frequency of 1.5 Hz, a force of 20 N, and a separation distance of 20 mm between the PVDF and nylon fabric counter-electrodes. The current output was measured using an electrometer (Keithley 6517B, Cleveland, USA), and the current and power response across external load resistances ranging from 0 to 1000 MΩ were also determined for an active area, *A* = 1.15 cm^2^. The peak-to-peak current values were analyzed using OriginPro (2022, OriginLab, USA). Finally, the current and power outputs were calculated by averaging results from three independent samples for both PVDF and 5 wt% BN/PVDF mats.

The triboelectric power output of PVDF Y and 5 wt% BN/PVDF Y was also evaluated in a contact separation mode using the same bespoke linear motor setup. To measure the generated triboelectric output, the yarns were continuously tapped against a nylon fabric using the linear motor. The active contact area of the yarn was ∼ 0.195 cm^2^ (considering the yarn as a cylinder). The nylon fabric was cut into a circular shape with a diameter of 24 mm and was affixed to an Al electrode using carbon tape. The tapping parameters were 1.5 Hz, 20 N, and a separation distance of 20 mm between the PVDF Y (or 5 wt% BN/PVDF Y) and nylon fabric counter-electrodes. For direct comparison, we also prepared rolled mat structures by tightly winding electrospun PVDF and 5 wt% BN/PVDF mats onto copper wires to form yarn-like samples, hereafter referred to as PVDF R and 5 wt% BN/PVDF R, respectively. These rolled samples were tested under the same tapping conditions (active area, *A* ≈ 0.246 cm^2^) to benchmark their performance against the spun yarns. The generated output current was recorded across various resistances (0 to 1000 MΩ) by an electrometer (Keithley 6517B, Cleveland, USA). The corresponding power outputs were calculated from current-resistance characteristics, and all values were the average of three different samples measured for each sample.

## Results and discussion

### Surface morphology and phase characterizations

**Fig. 1:**
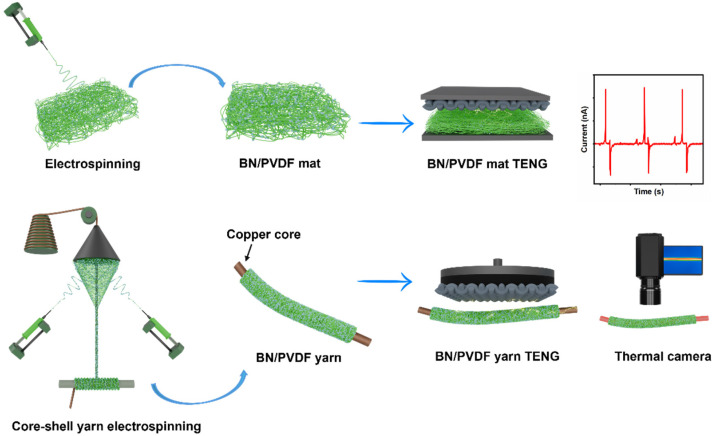
Conceptual schematic illustrating the fabrication of BN/PVDF composite mats and yarns and their triboelectric and thermal performance studied in this work.

To improve the functional performance of PVDF for energy harvesting applications, BN nanoparticles were incorporated into the PVDF matrix at various loading percentages (1, 3, 5, and 10 wt%). The main concept of this research is presented in Fig. 1. Electrospinning was employed to fabricate PVDF and BN/PVDF-based composite mats and yarns. The produced samples were labelled as PVDF M (for mat) and PVDF Y (for yarn) for pristine samples, and for BN composite samples, it is written as 1, 3, 5, and 10 wt% BN/PVDF M (for mats) and BN/PVDF Y (for yarns), respectively. In addition, yarn samples were prepared by rolling the electrospun mats to form a continuous yarn structure, specifically for PVDF and 5 wt% BN/PVDF compositions, to further assess their energy harvesting performance. These rolled mat samples were labelled as PVDF R and 5 wt% BN/PVDF R, respectively.

**Fig. 2:**
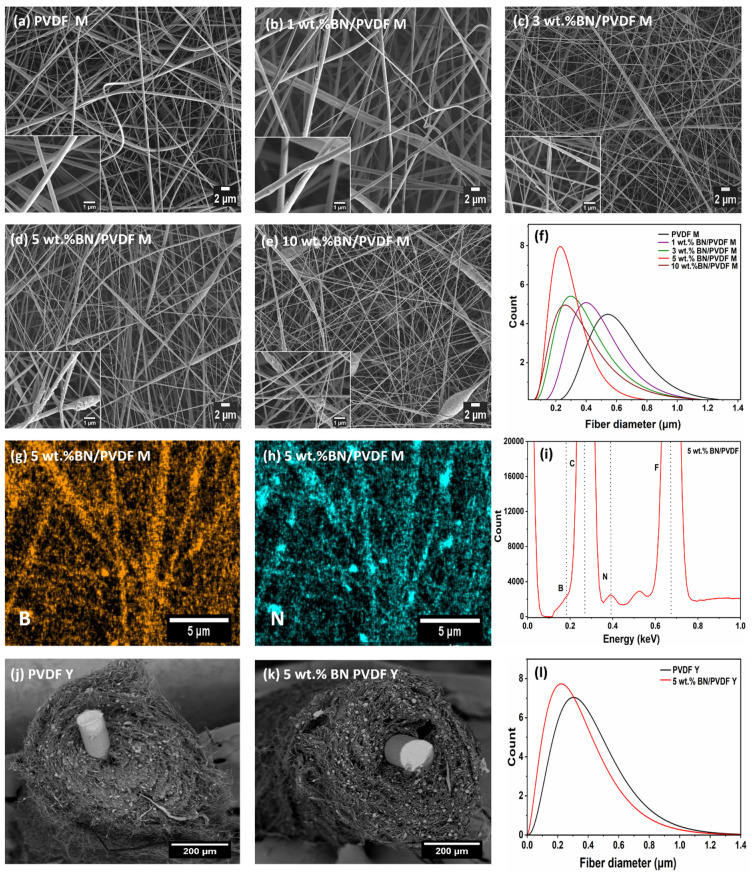
SEM micrographs of BN/PVDF composite mats and yarns (a) PVDF M (b) 1 wt% BN/PVDF M (c) 3 wt% BN/PVDF M (d) 5 wt% BN/PVDF M (e) 10 wt% BN/PVDF M. (f) Fiber diameter distribution curve from PVDF and BN/PVDF composite mats. (g–i) EDS elemental maps of B and N with the corresponding EDS spectrum. SEM micrographs of yarn cross-sections: (j) PVDF Y and (k) 5 wt% BN/PVDF Y yarns. (l) Fiber diameter distribution curve of PVDF and BN/PVDF yarns.

The morphology and microstructure of the PVDF and BN/PVDF composite fibers were investigated by SEM (Fig. 2). The SEM micrographs of electrospun mats are shown in Fig. 2a–e, where the high-magnification insets indicate the presence of BN nanoparticles on the surfaces of the individual fibers. The corresponding fiber diameter distribution curves for PVDF M and BN/PVDF M composites are presented in Fig. 2f. The pristine PVDF M exhibits bead-free fibers with an average diameter of 625 ± 187 nm. The average fiber diameter of 1 wt% and 3 wt% BN/PVDF M are 495 ± 193 and 423 ± 226 nm, respectively. However, the addition of 5 wt% BN into PVDF M significantly decreased the fiber diameter of PVDF M to 296 ± 121 nm due to the higher electrical conductivity of the solution containing nanoparticles.^47,48^ Conversely, further addition of 10 wt% BN in PVDF increases the aggregation of the BN nanoparticles, resulting in an increase in fiber diameter to 400 ± 235 nm (Fig. 2e). An increase in BN loading raises the solution viscosity, hindering uniform nanoparticle dispersion and reducing jet elongation during electrospinning. This results in agglomeration and the formation of thicker, bead-containing fibers.^49,50^ Therefore, the composite containing 5 wt% BN was identified as the optimal loading, as a higher content (10 wt%) led to beaded morphology (Fig. 2e), which is detrimental to effective filler-matrix interaction. The distribution of the nanofillers within the PVDF matrix was evaluated by EDS elemental mapping. As shown in Fig. 2g–i, the boron (B) and nitrogen (N) maps confirm the widespread distribution of BN at 5 wt%, while the corresponding spectrum verifies successful filler incorporation. The mapped region together with the carbon (C) and fluorine (F) distributions are provided in Fig. S2 (SI). Further, the yarns were produced by using copper wire/resistance as the core and electrospun fibers as the coating shell. The fabrication process utilized electrospinning with a specialized yarn module, in which nanofibers were uniformly deposited onto a copper wire and subsequently directed through a vortex collector for continuous yarn formation. The PVDF Y and 5 wt% BN/PVDF Y yarns wrapped around the copper wire are presented in Fig. 2j and k. The yarns exhibited a consistent alignment and twisting along the copper wire. Notably, the morphology of the nanofibers within the yarn structure closely resembled that of fibers obtained in mat form (see Fig. S3). Nonetheless, the average fiber diameter for PVDF Y (425 ± 233 nm) was smaller compared to that in the mat form (Fig. 2l). This reduction is attributed to the altered electric field distribution that occurs upon the introduction of the copper wire, which acts as an electrode and directs fiber deposition and alignment, thereby enhancing jet stretching and thinning through increased charge density and modified electrostatic interactions. For 5 wt% BN/PVDF yarn, the average fiber diameter slightly increased to 355 ± 221 nm compared to the mat, likely due to the formation of a more beaded fiber morphology.

**Fig. 3:**
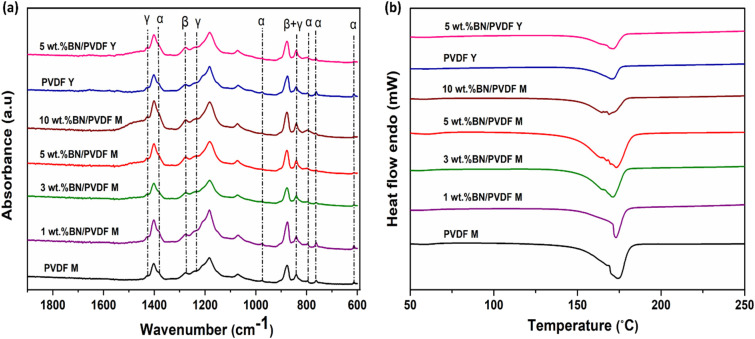
(a) FTIR spectra of BN/PVDF composites and (b) DSC heating thermograms of all samples.

The triboelectric performance of PVDF composite-based TENG is mainly dependent on the occurrence of the piezoactive phase content. Therefore, following the fabrication of PVDF and its composite fibers, the electroactive phases were investigated using FTIR. As shown in Fig. 3a, the characteristic bands at 610, 763 (CF_2_ bending), 795, and 977 cm^−1^ (CH_2_ twisting) are ascribed to the non-electroactive α phase of PVDF, in agreement with previous reports.^51–53^ In contrast, the band at 840 cm^−1^ is assigned to the β and γ crystalline phases and is associated with CH_2_ rocking and the asymmetric stretching vibration of the CF_2_ group.^53,54^ The peak at 1276 cm^−1^ is indicative of the electroactive β phase.^53,55^ In addition, the characteristic γ-phase bands observed at 1427 and 1235 cm^−1^ correspond to the asymmetric stretching of CF_2_ and the CH_2_ twisting vibration of PVDF.^52,53^

Compared to pristine PVDF M, all BN-incorporated composites exhibited an enhanced intensity of the β + γ phase peaks, suggesting increased electroactive phase formation. In PVDF M, α-phase peaks were prominent, while the composite samples showed a noticeable reduction or near disappearance of these α-phase signals (610, 763, 795, and 977 cm^−1^), indicating phase transformation. The relative β-phase fraction, *F*(β), was quantified using the method described in the literature.^43^ As summarized in Table S2, the incorporation of BN significantly increased the β-phase content. For example, the addition of 1 wt% BN increased the β-phase fraction from 67% in pristine PVDF to 76%. At 3 wt% BN, a sharp absorption band at 840 cm^−1^ (β + γ phase) was observed, and the β-phase content increased to 78%. This trend continued with higher filler concentrations, 5 wt% BN/PVDF M exhibited a β-phase fraction of 82%, while 10 wt% BN/PVDF M showed the same value of 82%. The enhanced β-phase content at increasing BN loadings can be attributed to the role of BN filler as a nucleating agent. The electronegative fluorine atoms in the –CF_2_ dipoles of PVDF can intermingle in dipole–dipole interactions with the polar B–N bonds of BN, thereby facilitating the preferential crystallization of PVDF.^56–58^ Similarly, in the PVDF Y sample, the β-phase fraction reached 71%, indicating a significant enhancement in the piezoelectric effect compared to typical PVDF mats. This improvement may be attributed to the mechanical stretching and alignment during yarn formation, which can favour β-phase orientation. Further enhancement was observed in the 5 wt% BN/PVDF Y composite, where the β-phase content increased to 79%. However, compared to 5 wt% BN/PVDF M, the β-phase content is relatively lower, this can be explained based on the more beaded morphology (Fig. S3b) of the fiber during yarn electrospinning. A comparative summary of the β-phase content of PVDF and PVDF-based composites reported in the literature and in this work is provided in Table S3.

DSC was employed to analyze the crystalline phases of PVDF mats and yarns, as well as to assess the influence of BN on the crystallization and melting behavior of electrospun PVDF fibers. The endothermic curves for PVDF M and BN/PVDF M composites are presented in Fig. 3b. Key thermal parameters, including melting temperature (*T*_m_) and degree of crystallinity (*χ*_c_), are summarized in Table S2. The crystallinity of each sample was calculated using a standard method reported in prior studies.^43,59^ As shown in Fig. 3b, all BN/PVDF composites exhibited higher crystallinity compared to pristine PVDF. The melting peaks observed in the range of 165–172 °C are attributed to the α-phase. The β-phase melting peak is overlapped with the α-phase, which appeared as a shoulder or as a broader peak.^57,60^ Here, the observed increase in melting temperature in the range of 173–174 °C for 3, 5, and 10 wt% BN/PVDF M can be ascribed to an enhanced γ-phase in the composite mats. Because γ-phase exhibits a higher melting temperature than the α- and β-phases.^57,60^ However, DSC is considered a complementary technique to distinguish the crystalline phases in PVDF. It does not provide direct proof to differentiate the crystalline phases due to their overlapping melting temperatures. Though it remains a reliable technique for determining the overall degree of crystallinity.^57,61^

The crystallinity of PVDF M was calculated to be 44.5%, and for 1 wt% BN/PVDF M was 44.1%. The crystallinity increased to 47.5%, 48.3% for composite fibers containing 3 and 5 wt% BN, respectively. The crystallinity decreased to 45.4 at 10 wt% BN, likely due to filler agglomeration and disruption of the polymer-filler interface at higher concentrations. PVDF Y exhibited a crystallinity of 42.7%, slightly lower than that of the PVDF mat. This may be attributed to differences in fiber morphology and processing conditions during yarn formation. In contrast, the addition of 5 wt% BN to PVDF Y resulted in a modest increase in crystallinity to 43.1%. This enhancement is attributed to the nucleating effect of BN nanoparticles, which facilitate crystal growth by providing heterogeneous nucleation sites.^59,62^ The marginal increase suggests that BN promotes crystallization.^62^

The characterization results confirmed that the inclusion of 5 wt% BN nanoparticle markedly increased both the β-phase and crystallinity of PVDF fiber and yarn. To further elucidate the influence of structural geometry and fabrication approach on thermal conductivity and triboelectric energy harvesting, we systematically evaluated mats, yarns, and rolled mat configurations. For this purpose, 5 wt% BN/PVDF M, 5 wt% BN/PVDF Y, and 5 wt% BN/PVDF R samples are selected and compared against their pristine counterparts, PVDF M, PVDF Y, and PVDF R.

### Thermal camera measurement of the yarn

**Fig. 4:**
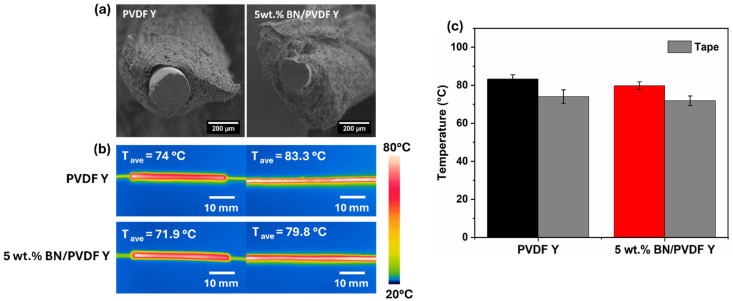
Overview of the morphology and thermal behavior of PVDF and 5 wt% BN/PVDF yarns (a). SEM micrographs of the cross-sectional view of PVDF and 5 wt% BN/PVDF yarns on the resistance wire. (b) Infrared images of standard tape on the left and the yarns on the right with the average temperature. (c) Column chart showing the average temperature of the yarns and the reference tapes.

To evaluate the thermal response, yarns were fabricated using a resistance wire as the core, subsequently coated with pristine PVDF and BN/PVDF composite layers. The resulting yarns (PVDF Y and 5 wt% BN/PVDF Y) are shown in Fig. 4a, exhibiting uniform alignment and twisting around the core, similar to the yarns prepared on copper wire (Fig. S3c and d). The average fiber diameters are in the range of 250–435 µm for PVDF Y and 5 wt% BN/PVDF Y. The thermal performance was assessed by connecting the resistance wire core to a controlled power source, adjusting the applied current such that the maximum surface temperature of the reference tape reached 80 °C, see Fig. 4b, showing the temperatures monitored by an infrared thermal camera. Fig. 4c indicates that the addition of 5 wt% BN nanoparticles does not significantly alter the surface temperature of PVDF. As shown in Fig. S4, the rolled mat samples exhibited thermal responses similar to those of the yarns, indicating that neither the geometric configuration nor the inclusion of BN nanoparticles had a notable effect on the surface temperature of the composites. This can be attributed to the relatively low filler content, which is insufficient to establish effective phonon transport pathways or a percolated thermal conduction network within the polymer.

Significant efforts have been devoted to overcoming the inherently low thermal conductivity of electrospun PVDF, with BN-based nanofillers being among the most effective solutions. The degree of improvement, however, is largely governed by the formation of continuous thermal pathways, which is influenced by filler concentration, dispersion quality, and fiber morphology. Zhang *et al*.^62^ fabricated electrospun PVDF composites incorporating modified boron nitride (m-BN) and reported a significantly enhanced thermal conductivity of 7.29 W m^−1^ K^−1^ at a 30 wt% filler loading. Similarly, Jin Zhang *et al*.^37^ introduced amino-functionalized BN nanosheets (BNNSs) into electrospun PVDF fibers and showed that 1.5% BNNSs yields forty times higher (6.5 W m^−1^ K^−1^) in-plane thermal conductivity than pristine PVDF fiber TENG.^63^ Further demonstrated a layered PVDF/BNNs composite film with 60 wt% BNNSs, attaining a remarkable thermal conductivity of 11.88 W m^−1^ K^−1^, nearly two orders of magnitude greater than pristine PVDF.

These studies collectively underscore that achieving substantial thermal conductivity enhancement requires either high BN loading or optimized filler alignment and interfacial compatibility to establish a continuous thermal conductive path. In contrast, low BN concentrations and high porosity in electrospun architectures hinder the formation of effective thermal networks.^46^ To address these limitations, strategies such as surface functionalization, post-processing treatments (*e.g.*, hot pressing), and hybrid electrospinning-electrospraying techniques have been employed to enhance thermal percolation.^46,64^

### Triboelectric measurement

We evaluated the triboelectric performance of BN/PVDF-based TENGs fabricated in three configurations: electrospun mats, yarns, and rolled mats. The devices operated *via* a contact–separation mode (CS) for mats and a single-electrode mode (SE) for yarns and rolled mats. Fig. S5a illustrates the schematic of the CS-TENG setup and its working mode. PVDF and BN/PVDF composites served as tribo-negative layers, while nylon fabric was selected as the tribo-positive counterpart to mimic realistic textile interfaces and demonstrate the practical applicability of the device in smart textile systems. Also, we fabricated pristine PVDF-based TENGs under identical conditions.

**Fig. 5:**
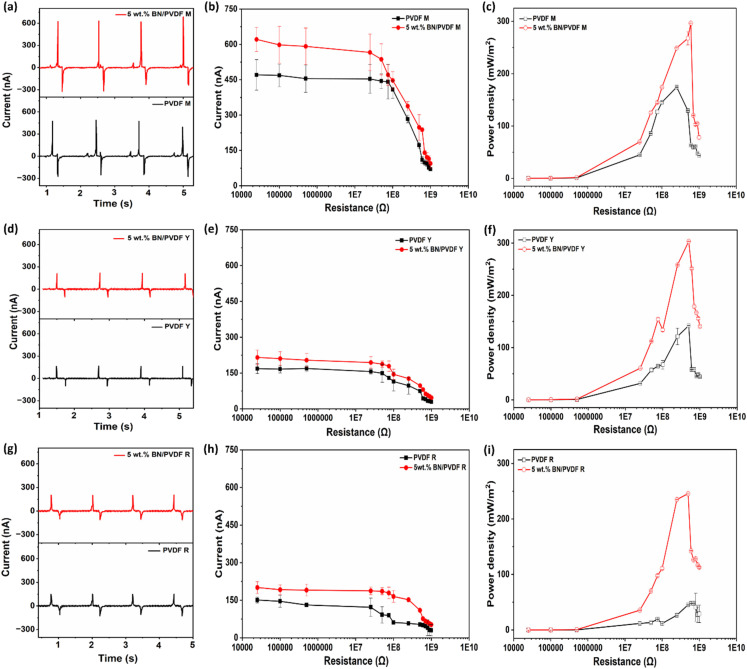
Performance comparison of PVDF and 5 wt% BN/PVDF TENGs fabricated in mat (M), yarn (Y), and rolled mat (R) geometries. (a–c) short-circuit current across 0 Ω, current output across different resistors, and power density for mat-based TENGs. (d–f) short-circuit current across 0 Ω, current output across different resistors, and power density for yarn-based TENGs (g–i) short-circuit current 0 Ω, current output across different resistors, and power density for rolled mat configurations.

Fig. 5a and b present the short circuit output current (*I*) profiles from PVDF M and 5 wt% BN/PVDF M, revealing that 5 wt% BN/PVDF M TENG delivers significantly enhanced performance relative to the pristine PVDF M device. The underlying charge transfer mechanism of CS-TENG can be explained as follows. When an external force is applied to the TENG mat, 5 wt% BN/PVDF M contacts the nylon fabric, generating opposite charges at the interface due to their distinct electron affinities. Electrons transfer from nylon to 5 wt% BN/PVDF M, leaving nylon positively charged and 5 wt% BN/PVDF M is negatively charged. During release, the resulting potential difference drives charge flow between the electrodes through the external load. The periodic contact and release cycles continuously break and restore electrostatic equilibrium, producing the triboelectric charge.^6,65^

Fig. 5a displays the short-circuit current output, where the 5 wt% BN/PVDF M exhibits a peak value of 622 ± 91 nA, outperforming pristine PVDF M (470 ± 61 nA). Fig. 5b presents the dependence of TENG performance on external load resistance (*R*, 0–1000 MΩ). With increasing resistance, the current decreased in accordance with Ohm's law, while the power density increased to its maximum at 250 MΩ for PVDF M and 600 MΩ for 5 wt% BN/PVDF M (the corresponding *I* measured across the resistance is presented in Fig. S6a). The power density was derived with varying load resistance from the equation *P* = *I*^2^*R*/*A* (where *A* is the active area of the device, 1.13 cm^2^). At this optimal load, the 5 wt% BN/PVDF M device delivered a peak power density of 297 ± 0.43 mW m^−2^, representing a ∼71% enhancement over pristine PVDF M (174 ± 0.54 mW m^−2^) (Fig. 5c). This enhancement is attributed to BN's role as an efficient charge-trapping site within the PVDF matrix, which promotes interfacial charge storage.^56,66^ BN, with its high piezoelectricity and large surface area, also contributes to converting mechanical energy into electrical energy.^2,58,67^ Zhang *et al.*^56^ demonstrated that incorporation of 2 wt% boron nitride nanosheets (BNNs) into PVDF fibers enhanced the triboelectric amplitude by nearly 600% relative to pristine PVDF, although power output was not reported. Similarly, Yang *et al*.^2^ demonstrated that introducing 0.5 wt% hexagonal BN nanosheets (hBNNSs) into PVDF mats significantly boosted the TENG performance, achieving a power density of 3.13 Wm^−2^. It should be noted that the TENG's active area (4 × 4 cm^2^) is larger than that employed in this study.

The SE mode operation of the yarn, illustrated in Fig. S5b, enabled a detailed investigation of the triboelectric output characteristics of BN/PVDF yarn. As shown in Fig. 5d, the pristine PVDF Y delivered an output current of 168 ± 20 nA, whereas incorporation of 5 wt% BN nanoparticles enhanced the current to 215 ± 30 nA, highlighting the beneficial role of BN in boosting charge generation. The dependence of current output on external load resistance was represented in Fig. 5e, where the BN/PVDF Y exhibits comparatively lower current than its mat counterpart, primarily due to the yarn's reduced effective contact area and less efficient electrode coupling, which limit charge transfer and collection.^68,69^ However, electrospun mats offer a large surface area, facilitating charge accumulation and transfer during repeated contact-separation cycles, and ensuring more uniform contact with the electrode layer.^6,65^Fig. 5f, the power density increased steadily with load resistance and reached a maximum at 500 MΩ and 5 wt% BN/PVDF Y achieved a peak power density of 303 ± 0.30 mW m^−2^, signifying an enhancement of ∼113% compared to pristine PVDF yarn (142 ± 0.24 mW m^−2^). The recorded current across the load resistance is shown in Fig. S6b. This substantial improvement is due to the synergistic role of BN nanoparticles in enhancing β-phase content and crystallinity, which promotes triboelectric energy generation.^2,67^ SE yarn devices exhibit a slightly higher power density (∼2%) than their mat counterparts, with a lower contact area than the mat. These findings confirm that BN/PVDF yarns are promising candidates for single-electrode TENGs, offering stable and enhanced energy harvesting capabilities.

When the electrospun mats were rolled around a copper wire to form a yarn-like configuration (PVDF R and 5 wt% BN/PVDF R), a pronounced reduction in triboelectric output was observed compared to both the pristine mat and the directly electrospun yarn architectures. The PVDF R and 5 wt% BN/PVDF R delivered output currents of 153 ± 11 nA and 196 ± 24 nA at 0 Ω, respectively (Fig. 5g), with further decreases across at higher external resistances (Fig. 5h). PVDF R obtained a peak power density of 48 ± 0.22 mW m^−2^, whereas the incorporation of 5 wt% BN enhanced the value to 246 ± 0.17 mW m^−2^ at 500 MΩ (Fig. 5i), indicating an increase of ∼412% (measured current across the load resistance is included in Fig. S6c). Despite this significant material-level improvement, the rolled geometry restricts performance relative to mats due to reduced effective contact area and poorer electrode contact, which limit charge transfer and power output.

Among the different structural geometries, the yarn configuration demonstrated superior triboelectric performance. Although yarns have a smaller geometric active area than planar mats, their cylindrical structure enables multidirectional contact during mechanical deformation, enhancing charge generation. Additionally, uniform nanofiber coating and optimized filler dispersion enhance surface interactions and charge transfer, enabling yarn-based devices to achieve high triboelectric performance despite reduced area.

**Fig. 6:**
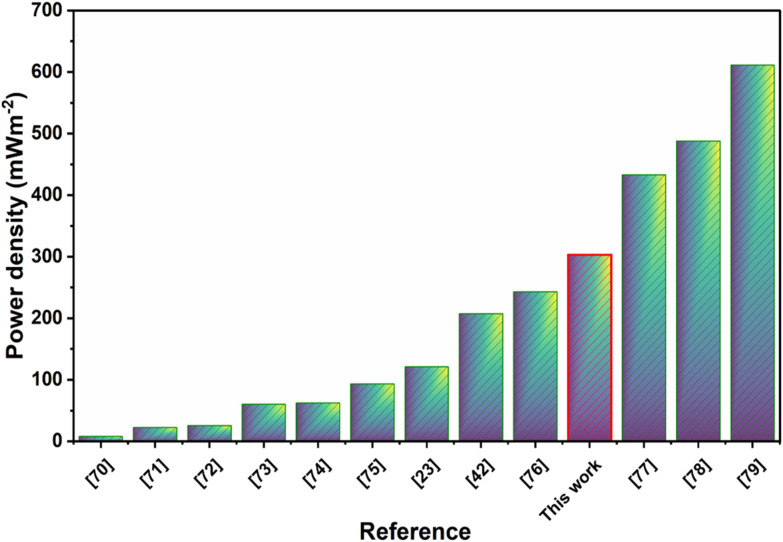
Literature comparison of triboelectric power density for yarn-based TENGs.

Mat and yarn-based TENGs have been developed with various structures and core–shell designs, nevertheless their output performances vary widely depending on device geometry, active area, and filler content, testing condition such as applied force, frequency and humidity. Several studies have demonstrated promising performance using different material systems and fabrication strategies. Fig. 6 illustrates the literature comparison graph exhibiting the maximum power density output from yarn based TENGs.^23,42,70–79^ A detailed comparison of the yarn fabrication methods and maximum power density of yarn-based TENGs reported in the literature and in this work is presented in Table S4. For instance, Busolo *et al*.^42^ reported a washable TENG comprising a CNT core and PVDF shell, achieving a peak power density of 207 mW m^−2^ even after 200 000 fatigue cycles. While Szewczyk *et al*.^23^ electrospun Nylon-11 on carbon yarns and demonstrated a power density of 121 mW m^−2^. Moradi *et al*.^77^ fabricated a multifunctional composite yarn based on polyacrylonitrile (PAN) and Ti_3_C_2_T_*x*_ MXene with a high MXene loading (50 wt%), coated on a copper wire, and reported a power density of 432.7 mW m^−2^. Similarly, Chen *et al*.^78^ developed a nano-microstructure yarn (NMSY) *via* a combination of conjugated electrospinning and ring spinning, in which Nylon-11 nanofibers were electrospun, and ring spinning of polyester fibers were wrapped as the outer layer. The resulting electronic textile exhibited a power density of 487.8 mW m^−2^. In another study, Ma *et al*.^79^ reported an ultralight nano-microfiber hybrid single-electrode triboelectric yarn (SETY) consisting of PVDF/PAN hybrid nanofibers as the shell and conductive silver yarns as the core, delivering a power density of 611 mW m^−2^. The BN/PVDF yarn developed in this work delivered a maximum power density of 303 ± 0.30 mW m^−2^ (Fig. 6) with a contact area of 0.195 cm^2^, placing it among the higher-performing electrospun yarn-based TENGs reported in the literature. This result highlights the effective role of BN incorporation in enhancing charge generation, despite the relatively small active area of the yarn structure.

Furthermore, all devices exhibited excellent operational stability, maintaining a consistent output performance over 2000 contact-release cycles (Fig. S6d and e), highlighting their durability and potential for long-term practical triboelectric energy harvesting applications.

## Conclusions

By uniting materials design and structural engineering, this work delivers a significant advancement in flexible energy-harvesting technologies. Through the strategic integration of BN nanoparticles into electrospun PVDF matrices, we unveil a highly effective route to enhance triboelectric performance. Electrospun BN/PVDF composite mats and yarns were systematically fabricated and examined to assess the effects of BN incorporation and structural geometry on triboelectric and thermal performance. The 5 wt% BN addition increased the β-phase fraction from 67% to 82%, confirming its role as an efficient nucleating agent, which contributes to improved electrical output. Furthermore, the thermal properties were studied using thermal camera imaging, and the results showed negligible variation in surface temperature between PVDF and BN/PVDF yarns, attributed to the low BN content, which is insufficient to form efficient thermal conduction networks. The triboelectric performance demonstrated that the 5 wt% BN/PVDF mat achieved a peak power density of 297 ± 0.43 mW m^−2^, representing a ∼71% improvement over pristine PVDF mats. Compared to the pure PVDF yarn, 5 wt% BN/PVDF yarn exhibited a higher output of 303 ± 0.30 mW m^−2^, corresponding to a ∼113% enhancement, despite its smaller active area. Therefore, the synergy between BN-induced electroactivity and yarn geometry offers a new design direction for multifunctional, textile-integrable energy harvesters, particularly relevant since mat structures cannot be directly woven or knitted into fabrics. Therefore, 5 wt% BN/PVDF offers a scalable and practical applicability in self-powered and wearable electronics.

## Author contributions

S. S.: conceptualization, methodology, experiment, characterizations, data analysis, writing – original draft, writing – review and editing, visualization. A. M.: experiment, review and editing. P. K. S.: review and editing, visualization. U. S.: conceptualization, resources, writing, review and editing, supervision, and funding acquisition.

## Conflicts of interest

There are no conflicts of interest to declare.

## Supplementary Material

NR-018-D5NR05178A-s001

NR-018-D5NR05178A-s002

## Data Availability

All data supporting the findings of this study are provided in the supplementary information. Supplementary information (SI): electrospinning parameters, a comparative summary of the β-phase content of PVDF based composite from literature, additional SEM images, thermal camera images, a schematic representation of yarn electrospinning, triboelectric bespoke setups, short-circuit current output graphs, and a comparison table of the power density of the BN/PVDF yarn in this work with previously reported yarn-based TENGs. See DOI: https://doi.org/10.1039/d5nr05178a. Additional datasets are available from the corresponding author upon reasonable request.
