# Supplementary material for: Flexible and efficient triboelectric nanogenerators based on PVDF and boron nitride composite yarns and mats
Source: Nanoscale. 2026 Jun 4;18(26):13793–804. doi: 10.1039/d5nr05178a (PMC13235273; doi:10.1039/d5nr05178a)
Supplement: NR-018-D5NR05178A-s001 [file NR-018-D5NR05178A-s001.pdf]

Supplementary Information (SI) for Nanoscale.
This journal is © The Royal Society of Chemistry 2026

Supplementary Information

Flexible and Efficient Triboelectric Nanogenerators based on PVDF and Boron Nitride Composite Yarns and Mats

Sunija Sukumaran, Ahmadreza Moradi, Piotr K. Szewczyk, Urszula Stachewicz*

Faculty of Metals Engineering and Industrial Computer Science, AGH University of Krakow 30-059 Krakow, Poland

Table S1. Electrospinning parameters for PVDF and BN/PVDF composite with different BN content at RH = 30 % and T = 25 ˚C.

Sample	BN content (wt.%)	Voltage (kV)	Distance (cm) (Needle tip to collector)	Flow rate (mlh^{-1})
PVDF M	0	20	18	1
1 wt.% BN/PVDF M	1	22	20	1
3 wt.% BN/PVDF M	3	22	20	1
5 wt.% BN/PVDF M	5	24	20	1
10 wt.% BN/PVDF M	10	26	20	1

Table S2. Summary of crystallinity and β phase content of BN/PVDF composite.

Sample	F(β) (%)	T_m (˚C)	χ_c (%)
PVDF M	67	169.02	44.5
1 wt.% BN/PVDF M	76	171.37	44.1
3 wt.% BN/PVDF M	78	173.07	47.5
5 wt.% BN/PVDF M	82	174.26	48.3
10 wt.% BN/PVDF M	82	173.42	45.4
PVDF Y	71	170.64	42.7
5 wt.% BN/PVDF Y	79	171.60	43.1

Table S3. Comparative summary of the β-phase content of PVDF and PVDF-based composites reported in the literature and in this work.

Material	Filler content (wt.%)	PVDF β phase fraction (%)	PVDF composite β phase fraction (%)	Authors' name
PVDF/ phenyl-isocyanate functionalized graphene oxide (IGO) nanofiber	0.05	68.7	97.1	Ramasamy et al.[1]
PVDF/GO nanofiber	2	~82	~87	Yang et al.[2]
PVDF/3Ag–P (metal phosphate nanostructure) film	3	68	96	Bahloul et al.[3]
PVDF/CNF fiber	0.5	86.9	93.2	Khadka et al.[4]
PVDF -TrFE/ Bismuth Ferrite composite film	6	80.2	83.7	Tripathy et al.[5]
PVDF/MWCNT-BaTiO3 electrospun fiber	15 wt.% BT and 0.15 wt.% MWCNT	77.45%	90.21	Lin et al.[6]
PVDF/Graphene fiber	0.1	77	83	Abolhasani et al.[7]
PVDF/MXene microinjected specimen	5	43.5	85.3	Han et al.[8]
PVDF-TiO_2 film	10	52	89	Kulkarni et al.[9]
Nitrogenous carbon dot/PVDF film	2.5% mass percent	~48	80.4	Sarkar et al.[10]
PVDF/BaTiO3	2	73.9	81.23	Kumar et al.[11]
PVDF-rGO-MoS_2 film		55	72	Faraz et al.[12]
PVDF/Silicon carbide film	6	-	81	Shafeek et al.[13]
PVDF/hBN nanofiber	0.4	~76	~86	Yadav et al.[14]
PVDF/BN nanosheet fiber	2	45	46	Zhang et al.[15]
PVDF/hBN film	1	62.2	85.4	Kumar et al.[16]
PVDF/BN electrospun fiber	5	67	82	This work
PVDF/BN Yarn	5	71	79	This work

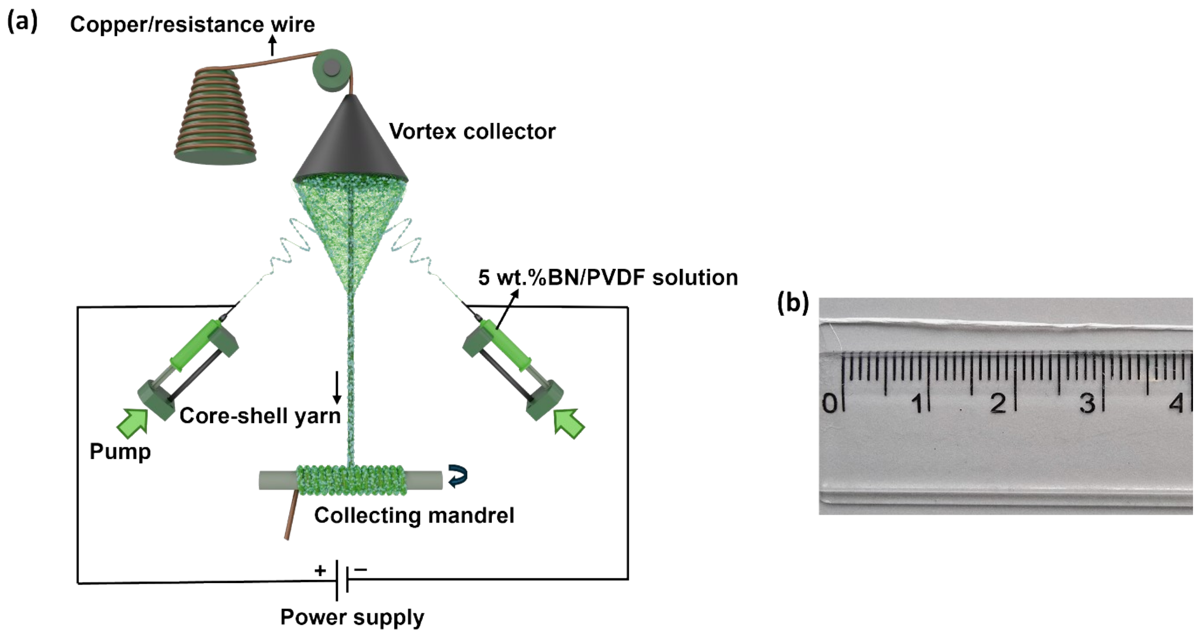

Fig. S1 (a) Schematic representation of the 5 wt.% BN/PVDF yarn fabrication process (b) Photograph of the produced 5 wt.% BN/PVDF yarn.

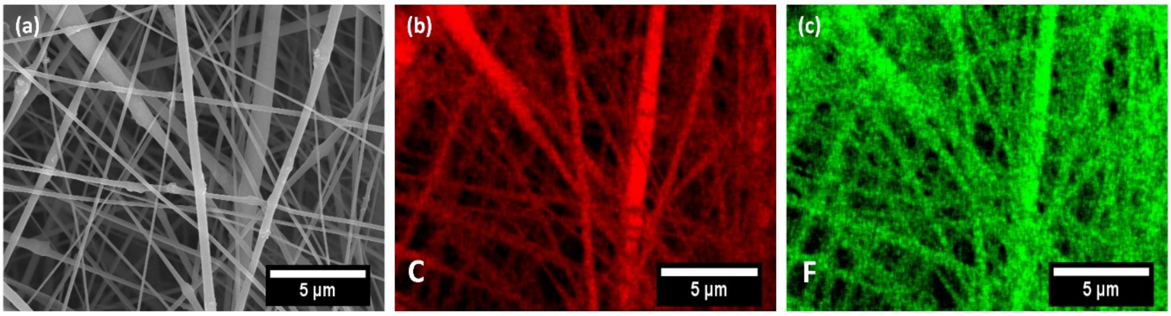

Fig. S2 (a) Schematic representation of the 5 wt.% BN/PVDF yarn fabrication process (b) Photograph of the produced 5 wt.% BN/PVDF yarn.

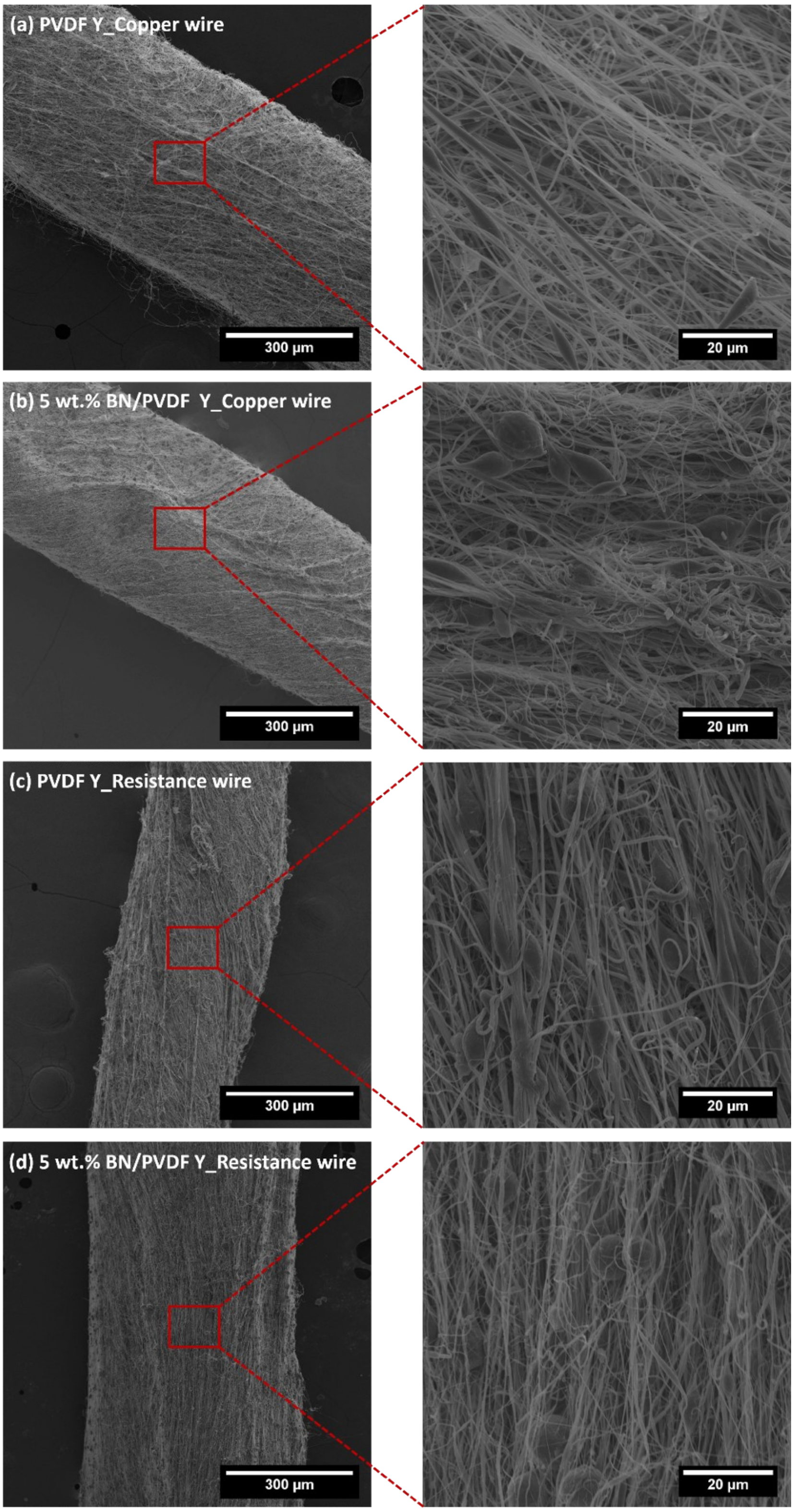

Fig. S3. SEM micrographs of the surface morphology of the yarn (left) and the higher magnification view of the fibers (right) for (a) PVDF Y on copper wire (b) 5 wt.% BN/PVDF Y on copper wire (c) PVDF Y on resistance wire (d) 5 wt.% BN/PVDF Y on resistance wire.

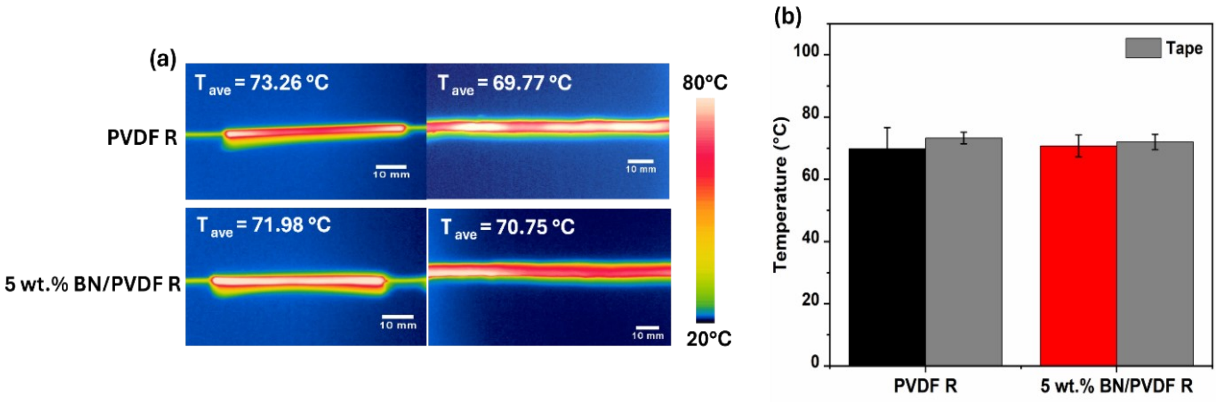

Fig. S4. (a) Infrared images of standard tapes and the rolled yarns with the corresponding average temperature (b) The column chart with the average temperature of the rolled yarns and the reference tape.

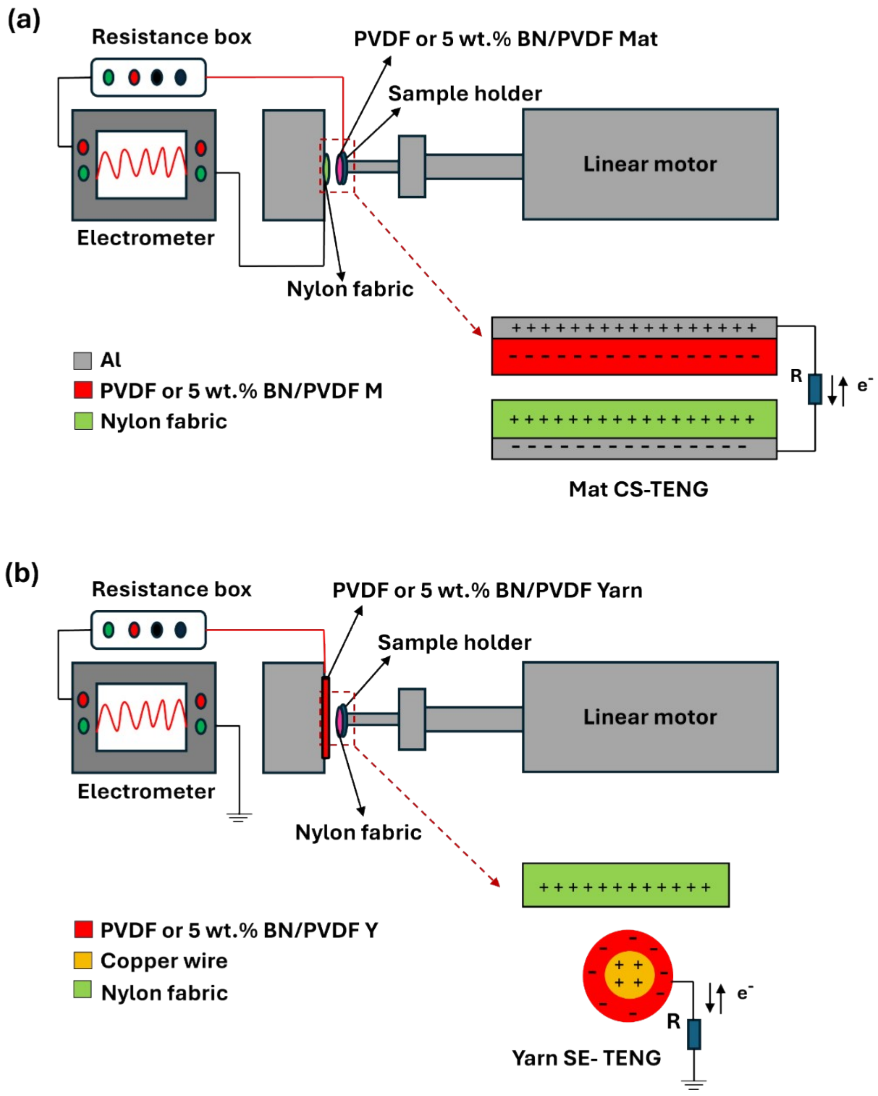

Fig. S5. Schematic representation of the triboelectricity testing system operating in different modes (a) Mat working in contact separation (CS) mode (b) Yarn working in single electrode (SE) mode.

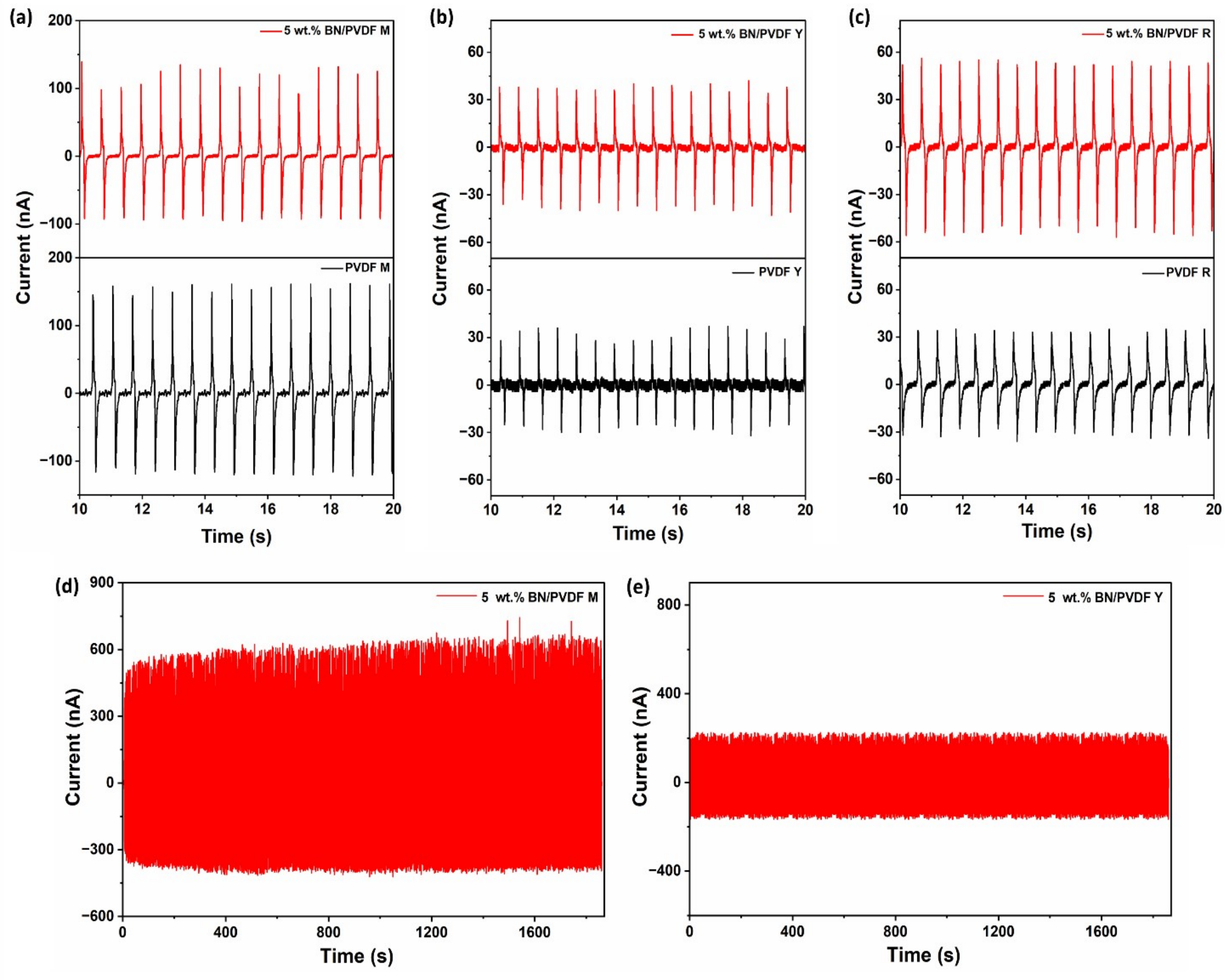

Fig. S6. Short-circuit current output corresponding to the load resistance at which the maximum power density was obtained for the TENG (a) PVDF M and 5 wt.%BN/PVDF M (b) PVDF Y and 5 wt.%BN/PVDF Y(c) PVDF R and 5 wt.%BN/PVDF R. Operational stability of the devices over 2000 contact-release cycles at ~0 Ω, demonstrating consistent output performance of mat and yarn (d)) 5 wt.%BN/PVDF M and (e) 5 wt.% BN/PVDF Y.

Table S4. Comparison of the power density of the BN/PVDF yarn in this work with previously reported yarn-based TENGs

**Material**	**Fabrication**	**Size**	**Power density (mWm^{-2})**	**Authors' name**
AgNW/MnO2-TPU layer and sheath-core TPU/CB@AgNW/PMMA	combining wet spinning with electrospinning, which was helically wound around the yarn-based ASC	1 cm in length and 0.5 cm in diameter	2.14	R. Tao et al. [17]
Core-shell yarn with core as conductive Ag-nylon yarn) and shell -PVDF-PTFE or PCL	Yarn electrospinning	3×3 cm	2.2	Zhou et al.[18]
Core - shell yarn with core as carbon yarn and shell-PA66-ZnO	Electrospinning on the carbon yarn	4×4 cm	2.7	Zamani et al.[19]
Core - shell yarn with core nickel-plated aramid yarn and shell FEP-doped FPI	Yarn electrospinning	2×2 cm	4.9	Hao et al.[20]
Core-shell yarn with core as nylon conductive yarn and shell PVDF-TrFE	Air-flow driven system (blow spinning)	2×4 cm	5.5	Chen et al.[21]
Core - shell yarn with core Ag yarn and shell-TPU	Direct electrospinning on the Ag yarn as the collector	5 cm in length and 1.3 mm diameter	5.6	Hu et al.[22]
Core-double shell yarn - core as conductive Ag-nylon yarn and shell 1 (PI) and shell 2 (PVDF-PDMS)	Yarn electrospinning	7.065 cm^2	7.7	Akram et al.[23]
Core-shell yarn with core copper wire and shell PVDF-Si3N4 and shell PU	Yarn electrospinning	5 cm x 5 cm	22.4	X. Tao et al.[24]
Core - shell yarn with core-carbon fiber and shell- PVDF-graphene	Yarn electrospinning	5 cm x 5 cm	25.5	Yang et al.[25]
Core-shell yarn - core stainless steal and shell-PU or Spandex	not electrospinning	4.5 cm x 8 cm	60	Yu et al.[26]
Core- shell yarn with core -stainless steel strands and shell (PA66) or (PVDF-TrFE)	Yarn electrospinning	-	62.1	Wang et al.[27]
Core-shell yarn - core stainless steal and shell-	Direct electrospinning and rolling	5 cm x 5 cm	93	Guan et al. [28]

PVDF-TrFE or shell PA66				
Core-shell yarn with core-CNT yarn and shell-PA11	Direct electrospinning on the CNT yarns as the collector	7.3 mm^2	121	Szewczyk et al.[29]
Core-shell yarn with core as carbon nanotube yarn and shell-PVDF	Direct electrospinning on the CNT yarns as the collector	0.096 cm^2	207	Busolo et al.[30]
Core-shell yarn with core-PTFE twisted with carbon fiber and shell PI + SiO2	yarn electrospinning (single nozzle)	-	243	Xing et al.[31]
Core-shell yarn with core as copper wire and shell 5 wt.% BN/PVDF	Yarn electrospinning	0.195 cm^2	303	This work
Core-shell yarn with core as copper wire and shell 50 wt.% $Ti_3C_2T_x$ MXene nanoflake/PAN composite	Yarn electrospinning	2.4 cm in length and 0.238 mm diameter	432.7	Moradi et al.[32]
Core-double shell yarn-core as copper yarn and shell1 (PA11 + ZnO) and shell2 (polyester fibers)	Combination of electrospinning and ring spinning	2.5 cm x 2.5 cm	487.8	Chen et al.[33]
Core-shell yarn - core as conductive silver yarn and shell- hybrid PAN/PVDF	Yarn electrospinning	1.375 cm^2	611	Ma et al.[34]

References

1 M. S. Ramasamy, A. Rahaman and B. Kim, *Ceramics International*, 2021, **47**, 11010–11021.

2 J. Yang, Y. Zhang, Y. Li, Z. Wang, W. Wang, Q. An and W. Tong, *Materials Today Communications*, 2021, **26**, 101629.

3 C. Bahloul, A. Eddiai, O. Cherkaoui, F.-Z. Semlali, M. El Achaby, and others, *RSC advances*, 2025, **15**, 15218–15239.

4 A. Khadka, E. Samuel, B. Joshi, A. Aldalbahi, G. Periyasami, H.-S. Lee and S. S. Yoon, *Nano Energy*, 2025, **139**, 110991.

5 A. Tripathy, N. P. M. J. Raj, B. Saravanakumar, S.-J. Kim and A. Ramadoss, *Journal of Alloys and Compounds*, 2023, **932**, 167569.

6 X. Lin, F. Yu, X. Zhang, W. Li, Y. Zhao, X. Fei, Q. Li, C. Yang and S. Huang, *ACS Applied Nano Materials*, 2023, **6**, 11955–11965.

7 M. M. Abolhasani, K. Shirvanimoghaddam and M. Naebe, *Composites Science and Technology*, 2017, **138**, 49–56.

8 R. Han, L. Zheng, G. Li, G. Chen, S. Ma, S. Cai and Y. Li, *ACS applied materials & interfaces*, 2021, **13**, 46738–46748.

9 N. D. Kulkarni and P. Kumari, *Materials Research Bulletin*, 2023, **157**, 112039.

10 D. Sarkar, N. Das, M. M. Saikh, P. Biswas, S. Roy, S. Paul, N. A. Hoque, R. Basu and S. Das, *Ceramics International*, 2023, **49**, 5466–5478.

11 M. Kumar, N. D. Kulkarni and P. Kumari, *Materials Research Bulletin*, 2024, **174**, 112739.

12 M. Faraz, H. H. Singh and N. Khare, *Journal of Alloys and Compounds*, 2022, **890**, 161840.
13 S. Shafeek, N. T. Balakrishnan, B. Fatma, A. Garg, D. Morton, J. Luo, P. Raghavan, and others, *Nano Energy*, 2023, **107**, 108146.
14 P. Yadav, T. D. Raju and S. Badhulika, *ACS Appl. Electron. Mater.*, 2020, **2**, 1970–1980.
15 J. Zhang, H. Wang, P. Blanloeuil, G. Li, Z. Sha, D. Wang, W. Lei, C. Boyer, Y. Yu, R. Tian and C. H. Wang, *Composites Communications*, 2020, **22**, 100535.
16 A. Kumar, S. Sharma, M. Jayasimhadri and N. K. Puri, *Journal of Materials Science: Materials in Electronics*, 2026, **37**, 98.
17 R. Tao, Y. Mao, C. Gu and W. Hu, *Chemical Engineering Journal*, 2024, **496**, 154358.
18 M. Zhou, F. Xu, L. Ma, Q. Luo, W. Ma, R. Wang, C. Lan, X. Pu and X. Qin, *Nano Energy*, 2022, **104**, 107885.
19 M. Zamani, A. Valipouri, S. A. H. Ravandi and A. Alsikh, *Energy Technology*, 2024, **12**, 2400687.
20 M. Hao, X. Hu, Z. Chen, B. Yang, Y. Liu, Q. Wang, X. Gao, Y. Liu, X. Wang and Y. Liu, *ACS Applied Materials & Interfaces*, 2024, **17**, 1038–1048.
21 Y. Chen, J. Hua, Y. Ling, Y. Liu, M. Chen, B. Ju, W. Gao, A. Mills, X. Tao and R. Yin, *Chemical Engineering Journal*, 2023, **477**, 147026.
22 S. Hu, Z. Ge, M. Chen, P. Ding, W. Zhai, G. Zheng, K. Dai, C. Liu and C. Shen, *Chemical Engineering Journal*, 2025, **510**, 161794.
23 W. Akram, Q. Chen, X. Zhang, S. Ren, L. Niu and J. Fang, *Nano Energy*, 2024, **131**, 110275.
24 X. Tao, Y. Zhou, K. Qi, C. Guo, Y. Dai, J. He and Z. Dai, *Journal of Colloid and Interface Science*, 2022, **608**, 2339–2346.
25 T. Yang, C. Wan, X. Zhang, T. Liu, L. Niu, J. Fang and Y. Liu, *Nano Res.*, 2024, **17**, 4478–4488.
26 A. Yu, X. Pu, R. Wen, M. Liu, T. Zhou, K. Zhang, Y. Zhang, J. Zhai, W. Hu and Z. L. Wang, *ACS nano*, 2017, **11**, 12764–12771.
27 Y. Wang, L. Chu, S. Meng, M. Yang, Y. Yu, X. Deng, C. Qi, T. Kong and Z. Liu, *Advanced Science*, 2024, **11**, 2401436.
28 X. Guan, B. Xu, M. Wu, T. Jing, Y. Yang and Y. Gao, *Nano Energy*, 2021, **80**, 105549.
29 P. K. Szewczyk, T. Busolo, S. Kar-Narayan and U. Stachewicz, *ACS Appl. Mater. Interfaces*, 2023, **15**, 56575–56586.
30 T. Busolo, P. K. Szewczyk, M. Nair, U. Stachewicz and S. Kar-Narayan, *ACS Appl. Mater. Interfaces*, 2021, **13**, 16876–16886.
31 F. Xing, Z. Ou, X. Gao, B. Chen and Z. L. Wang, *Advanced Functional Materials*, 2022, **32**, 2205275.
32 A. Moradi, P. K. Szewczyk and U. Stachewicz, *Advanced Materials*, 2025, e22098.
33 W. Chen, W. Fan, Q. Wang, X. Yu, Y. Luo, W. Wang, R. Lei and Y. Li, *Nano Energy*, 2022, **103**, 107769.
34 L. Ma, M. Zhou, R. Wu, A. Patil, H. Gong, S. Zhu, T. Wang, Y. Zhang, S. Shen, K. Dong, and others, *ACS nano*, 2020, **14**, 4716–4726.
